# On the “Summer Complaint” of Infants

**Published:** 1880-08

**Authors:** J. Lewis Smith

**Affiliations:** Clinical Professor of Diseases of Children in the Bellevue Hospital Medical College, New York


### ON THE "SUMMER COMPLAINT" OF INFANTS. A Clinical Lecture. By J. LEWIS SMITH, M.D., Clinical Professor of Diseases of Children in the Bellevue Hospital Medical College, New York.

GENTLEMEN: The hot weather is now approaching, during which the most prevalent and fatal disease of the cities is the so-called "summer complaint" of infants. It is espcially fatal in the large cities of the Atlantic coast. It is an intestinal catarrh, which begins to appear in the middle and latter part of May, and becomes more and more frequent, reaching its maximum prevalence and severity in mid-summer, after which new cases are less and less frequent till the last of October, when the epidemic gradually disappears, and no cases remain except such as have lingered from the hot months.

The "summer complaint" is, anatomically, a catarrh of the lower part of the small intestine, and of the entire large intestine: the inflammatory lesions being, in fatal cases, most marked in the descending portion of the colon, and especially in the sigmoid flexure, where the excrementitious matter is most apt to be delayed and to accumulate. The greater thickening and redness of the mucous membrane in this locality, which I have often observed at autopsies, has seemed to me chiefly due to the irritating nature of the fecal matter, just as the erythematous redness of the skin around the anus present in certain cases, arises from this cause. The ulcerations which are apt to occur in grave protracted cases, and which are circular and seem to correspond in site with the solitary glands, are also most numerous in the descending colon, and particularly in the sigmoid flexure. Most cases of this disease occur prior to the age of two and a half years; so that the "summer complaint" is almost peculiar to infancy. But the first years of childhood are not entirely exempt.

There are two important factors in the causation of the "summer complaint:" 1st. The atmospheric heat, which acts as a causative agent, not so much by its direct effect on the system—

though its direct enervating influence may act to a certain extent, as a cause, by impairing the general tone of the system, and the digestive function in particular—as by producing foul gaseous exhalations from the decaying organic matter, which always exists in considerable quantity in and around the crowded domiciles of the poor in our cities. Abundant observations show that the "summer complaint" is most frequent and fatal in those localities, where the streets, court-yards and apartments are most filthy. In these places the foul odors are often very perceptible to the visitor. The operation of this cause seems to me to be similar to the foul air of the dissecting-room, in former times, when the medical student remaining long at dissection was apt to be incommoded by attacks of diarrhœa.

2d. The other cause is not less important, and in a large proportion of cases its action is apparent. It is the use of unsuitable food. For food which the feeble digestion of the infant cannot assimilate, soon begins to undergo fermentive changes, and acts as an irritant and purgative. It is the common opinion among families that the "summer complaint" is most frequent and dangerous in the second summer, but many observations establish the fact, that the first summer possesses no immunity, but on the other hand, the younger the infant, the greater the liability to be attacked if the diet is improper, and the reasons why so many infants remain well in the first summer is that they are nourished at the breast, and this second factor or cause is not operative in them.

It is not my intention at this time to speak at length of measures designed to prevent the "summer complaint," or of the hygienic measures required in its treatment' except to state certain facts, which are very important, and the knowledge of which will materially assist the physician who has had little experience with the disease. First, every infant under the age of twelve months in the city should, if possible, have the breast milk during the hot season. If the mother be not competent to furnish it, a wet-nurse should be employed. If it cannot have the breast milk, the family should be urged to go to the country, especially during July and August. Never consent to the weaning of an infant in or just before the hot weather. Statistics which I have preserved, bearing on this point, show a most disastrous consequence. The new diet will not agree, and if weaning be necessary, insist on removal to the country.

But there is a large number of infants in the families of the city poor that are weaned at this improper time, no physician having been consulted, and a large number also who are wet-nursed, but the breast-milk is insufficient, and it is necessary to employ more or less artificial food. Now it is very important to decide what kind of food should be given. The shops contain several so-called "substitutes" for breast milk, but the milk of the cow or goat more closely resembles human milk, than do any of these "substitutes," and when sufficiently fresh, and of good quality, it is to be preferred, I think, for most infants. For an infant under the age of two months it should be diluted with one-third its quantity of water; for those between the ages of two and five months, one-fourth or one-fifth its quantity of water should be added; while for those over the age of five months, no dilution is ordinarily required. The infant after the age of one month should not take the breast more frequently than every second hour, and for artificial feeding it is well to have a little longer interval.

The casein of cow's or goat's milk is apt to coagulate in the stomach of the young infant in large masses, which are with difficulty digested. To aid, in a measure, in preventing this a thin farinaceous food, as barley-water, or rice-water well boiled, may be mixed with the milk. The farinaceous particles intimately mixed with the casein tend mechanically to prevent the coagulation in large masses. In an infant now under observation in the New York Infant Asylum, cow's milk given in the ordinary way caused vomiting, but, by preparing from it a wine whey, with a small proportion of sherry wine by which a considerable part of the casein is removed, it is no longer troubled with indigestion and is doing well. The employment of fresh wine whey, thus prepared, will often be found useful, at least as a temporary expedient, when the infant vomits milk prepared in the ordinary manner, and containing the full amount of casein. It has been proposed by Drs. Rudisch and Jacobi of this city to coagulate the casein, or at least a part of it, by hydrochloric acid before the milk is used. A half teaspoonful of the dilute officinal acid is added to one pint of cold water, and this is thoroughly mixed with two pints of cold milk, and the mixture is then boiled ten or fifteen minutes. This has been employed for only one infant under my observation, in one of the institutions. It had vomited the cow's milk previously given, and it vomited this as well. I design, however, to give the acidulated milk a more extended trial in the approaching hot season.

Another way of getting rid of a considerable part of the casein, which I have for many years employed, is to allow the milk, as soon as received, to stand for two or three hours, when the casein, from its greater specific gravity, has a tendency to fall, and the cream to rise. The upper portion can then be removed for use. If, as I think is ordinarily the case, this gives too much butter, the superficial layer of cream can be separated and rejected.

Still, there are many infants during the summer months, with whom cow's milk prepared in the usual way does not agree, and will not be likely to agree, however prepared, and condensed milk is found to do no better. For such, a thin gruel made from barley, rice, or wheat flour (the last having by preference been boiled in a dry state, several hours in a bag, and then grated) should be mixed with the milk in equal quantity, or the gruel may be in excess. By such aids we do the best we can to assist the feeble digestive function of the infant, but nothing which we can prepare will take the place of human milk for those under the age of twelve months. Of the various kinds of infant's food found in the shops, Ridge's food, prepared with milk, and Nestle's lacteous farina, prepared with water, have been favorably received, and many infants do well upon them except in the hottest weather. The medical profession will always regard with kindly feelings, the earnest endeavor of the renowned chemist, Baron Liebig, in the last years of his life to prepare a substitute for human milk, so important did he consider the subject of infant diet. Physiology at that time taught that young infants could digest only a very small quantity of starch, since in the first months of life the amount of salivary secretion by which starch is converted into glucose, a necessary change in digestion, is inconsiderable. But now it is ascertained that there is an epithelial ferment, which effects this change as well as the saliva. As Prof. Flint, Jr., says " * * * the intestinal juice of itself is capable of effecting the transformation of starch into sugar to a considerable extent," and an epithelial ferment in the buccal cavity appears to act in the same way (Richet). Therefore it seems that the youngest infant can digest a moderate amount of starch, though farinaceous substances are not to be depended on as a chief article of diet for any infant under the age of four or five months.

But the theory that starchy food could not be digested, or is digested slowly and insufficiently by such infants led Liebig to the preparation of his food, in which starch is converted into glucose by the action of malt. Now I have used quite exten-tensively the three preparations of Liebig's food contained in the shops, namely Hawley's, Horlich's, and Mellin's, and although they do well, answering the purpose for which they were designed, in the spring, autumnal, and winter months, I have discarded their use—in the summer season. If employed, except in small quantity, they certainly produce a laxative effect, from the large quantity of grape sugar which they contain, during those months when there is special liability to diarrhœal attacks. With these preliminary remarks, I wish to speak more particularly of the medicinal treatment of summer complaint.

While it is very important that this disease should receive early treatment, unfortunately many cases are allowed to run on for days even weeks before the physician is called. This neglect I find to be due in many instances to the belief common in the community, that a relaxed state of the bowels renders dentition less dangerous and is a relief to it, so that the infant may have half a dozen alvine discharges each day, and the parents believe that they are salutary, till finally they are alarmed by the evident loss of flesh and strength.

As in many other maladies, the medical treatment has materially changed within the last few years. Calomel, formerly administered in small doses in belief that the function of the liver needed rectifying,—this belief arising from the fact that the stools are green in many cases,—has been discarded. The green color is now known to be produced in the intestines at a considerable distance below the point where the bile enters it, and to be the result apparently of the admixture of the intestinal secretions with the fecal matter, just as the stools sometimes have the normal color when evacuated and become green from the action of the urine. The old dread of administering opiates to infants, promoted in this country by the perusal of Beck's "Infant Therapeutics," has ceased, in consequence of accumulated observations, showing their good effects, and the little risk which attends their judicious employment. The vegetable astringents, such as kino, catechu, and tannin, are now little used; another agent which is more readily administered, less likely to cause nausea and more effectual, taking their place.

Occasionally it is proper to commence treatment by the administration of a gentle purgative, as a single dose of castor oil, or a mixture of syrup of rhubarb and castor oil, when there is reason to think that the diarrhœa has been excited or aggravated by some irritating substance which the infant has swallowed, and which should be expelled before measures to restrain the stools are employed; but ordinarily no such preliminary treament is required, as the diarrhœa has contiued so long when the physician is called that any fruits or other substances injudiciously given must already have been expelled, or the history of the case shows that no such substance has been taken.

Our main reliance must be on opium and bismuth subnitrate, for the purpose of checking the stools and arresting the catarrh, and, as fermentative changes in the milk usually cause an excess of acids in the intestines, on some alkali, as the preparations of chalk. Opium is useful in checking the intestinal catarrh of infants as it is in that of adults, but I seldom give it or other medicine in the form of powder, experience having taught me that powders are apt to be partly wasted.

For infants under the age of five months opium may be safely and conveniently administered in paregoric, in doses of three drops to an infant of one month, five drops to one of three months, and eight drops to one of five months. For an infant of six months the deodorized tincture of opium should be given in half-drop doses, and for one of twelve months in drop doses. The interval between doses should be about three hours. Opium should never be prescribed in those advanced or grave cases in which there is marked drowsiness, or rolling of the head due to incipient spurious hydrocephalus. Refraining from its use for such patients, and giving it in the doses and with the interval stated above, I have almost never had occasion to regret its employment, though prescribing it several times each day during the hot weather, in private and asylum practice. Drops are of very variable size when falling from the mouth of a bottle, and the dropper should always be employed, so that the drop may be as nearly as possible half a minim. The metric system insures more exactness, but we are not yet ready for it in this country. In certain severe cases the opiate may be given for a time every two hours.

The bismuth subnitrate is applicable to all cases, possessing no disadvantanges, and producing no ill effects to limit its use. It may be given safely and with advantage in all stages of the malady as long as there is vomiting or diarrhœa. It is an efficient anti-emetic and antiseptic. It seems to act mainly locally, restraining the stools, increasing their consistence, and producing a soothing and curative effect upon the inflamed surface. It probably retards fermentative changes. It becomes a bismuth sulphide as dark as charcoal in the stomach, as I have observed at autopsies, and if taken in large doses, as it should be, it renders the stools dark. It sometimes taints the breath like that from onions or garlic, but I am informed that this is probably due to some impurity. It has long been in use in practice, but in insufficient and inadequate doses, for infants, until within recent years. An infant of one year should take it in doses of ten or twelve grains, and one of six months in six or eight grains.

Although we do not wish to produce alkalinity within the stomach, as the pepsin in its normal state is acid, and necessarily so for healthy digestion, yet in the "summer complaint" there is an undue development of acids, probably mainly the lactic. Therefore akalies, as lime-water and the preparations of chalk, have long been used in this disease, and with apparent benefit, especially in the more acute cases. Alcohol, in moderate quantity, aids digestion, and is a heart stimulant. It is quickly eliminated from the system of the infant, and should be given at intervals of one to two hours to those who require it. It is given best in the form of Bourbon whisky, brandy, or sherry, port, or Madeira wine. It is urgently demanded when, from failing heart action, hypostatic congestion of the lungs or brain is occurring or impending. In spurious hydrocephalus, which complicates many severe and protracted cases, its timely use aids materially in averting the danger. Many years ago Dr. Gooch, of London, was in the habit of employing in such cases of failing heart action the aromatic spirits of ammonia, and in five-drop doses sufficiently diluted in water it is a good stimulant and antacid.

Mild cases do not require special treatment designed to reduce temperature, as it is but moderately elevated. If the heat of the head be moderately increased, a cloth wrung out of cold water should be constantly applied to it, and especially should the nurse be forbidden to cover the head except in the slightest and coolest manner, either in the house or out-door. In those severe cases known as choleriform diarrhœa, or cholera infantum, more energetic anti-pyretic local treatment is required. This form of intestinal catarrh, which usually occurs quite abruptly, having been preceded either by a state of health, or mild diarrhœa, and is characterized by frequent watery stools and rapid loss of flesh and strength, is attended by a temperature unusually high, namely from 104° to 106°, or even higher. Such great elevation of temperature involves danger, and the prompt use of measures designed to reduce it constitutes an important part of the treatment. A bladder containing ice may be applied to the scalp, separated from it by one or two thicknesses of muslin, and the excessive heat of the hands and arms may be reduced by frequent sponging with cool water, and clysters of iced starch-water or mucilage are proper.

To these general observations in reference to the selection and use of therapeutic agents, it may be useful to the young practitioner to add certain formulæ, which, or the equivalents of which, experience has shown to be useful. To control the diarrhœa the following should be prescribed for an infant of one year: ℞. Tinct. opii deodorat. gtt. xvj ; bismuth subnitrat. ʒij ; syr. simplic. ℥ss ; mistur. cretæ, ℥iss. Misce. Shake bottle and give one teaspoonful every three hours. An infant of six months can take half the dose. A powder of bismuth, with the compound powder of chalk and opium, or with Dover's powder, has a similar effect.

Vomiting is often a very troublesome symptom, but the bismuth in the above prescription not unfrequently checks it. Its cause is probably not always the same. It is sometimes relieved by lime-water and brandy in milk when due to an excess of acid in the stomach, but the following prescription has given me more satisfaction than any other: ℞. Vini ipecacuanhæ, gtt. ij; bismuth subnitrat, ʒij; syr. zingiberis, aq. menth. peperitæ, āā ℥j. Misce. Shake the bottle and give one teaspoonful whenever there is nausea.

Whenever there are tenesmus or streaks of blood in the stools, and in other cases which are exceptionally obstinate, the following will be found useful as a clyster; ℞. Argent. nitratis, gr. j; bismuth subnitratis, ℥ss; mucil. acaciæ, aquæ, āā ℥ij. Misce. The whole of it or a part may be given through a glass or gutta-percha syringe, and retained for half an hour or one hour by a compress. Towards the close of the hot weather and in fall months, we are often asked to examine and treat protracted cases, that have been gradually losing flesh and strength since the diarrhœa began several weeks previously. The febrile movement is usually moderate, the appetite poor, the stools are not healthy looking, and probably less frequent than at first, though still too frequent. For such cases the following should be prescribed, which is both tonic and astringent: Liq. ferri nitratis, gtt. xxvij; tinc. colombæ, ʒiij; syr. simplic. ℥iij. Misce. Give a teaspoonful every two or three hours to an infant of one year. The beef wine and iron of the shops, of late extensively employed in New York as a general tonic, also has a good effect, for the iron which it contains is mildly constipating. The dose of it is also one teaspoonful for an infant of one year.

Quinia has not seemed to me to act well in diarrhœal affections of infancy, apparently increasing the number of stools. It should never be used in acute cases. If ever admissible, it is in the chronic and wasted cases as a tonic, and not for its anti-pyretic effect.

The tincture of digitalis as a heart tonic, in cases of impending or established spurious hydrocephalus or hypostatic pneumonia, is a good adjuvant. It should be given in one-drop doses every third hour, while more active stimulation by the carbonate or aromatic spirits of ammonia is employed. Digitalis will also probably be found useful in any case attended by weak and rapid pulse, even when there are no symptoms of the complications alluded to.

I have said nothing in reference to the use of pepsin and lactopeptine, for I have never been able to satisfy myself to what extent, or whether they materially assisted the digestive function in this disease. Experiments show that the quantity of food which either one in ordinary medicinal quantity digests is very small.

---

A NEW REMEDY FOR HYDROPHOBIA.—M. Lesserteur. *Journ. de Méd.*, May 1880.—The author makes known a plant which enjoys a great reputation for the cure of hydrophobia in the kingdom of Annam. This plant, called hoang-nan, is a species of convolvulous, which is closely related to false angustura; its effects should be analogous to those of strychnia and brucine. M. Bouley, in speaking of this new remedy in the *Recueil de Méd. Vétérinaire*, regrets that no case has been cited demonstrative of its efficacy; but thinks that experiments sho ıld be made upon the rabbit, which readily contracts hydrophobia by inoculation. M. Bouley relates an anecdote apropos of garlic, a substance which enjoys a great reputation among anti-hydrophobic remedies, and which is an ingredient of a good many formulæ long kept secret. A young man had been bitten by a mad dog and symptoms of hydrophobia soon manifested themselves. The affrighted family, not knowing what to do with the unfortunate, shut him up in a small chamber in which garlic had been placed to dry. In his fury the unfortunate man threw himself upon the bundles of garlic, ate of them abundantly, and soon became drowsy and fell into a profound sleep. On awakening he was cured; the symptoms of hydrophobia had entirely disappeared.

